# Stepwise modifications of genetic parts reinforce the secretory production of nattokinase in *Bacillus subtilis*

**DOI:** 10.1111/1751-7915.13298

**Published:** 2018-07-08

**Authors:** Wenjing Cui, Feiya Suo, Jintao Cheng, Laichuang Han, Wenliang Hao, Junling Guo, Zhemin Zhou

**Affiliations:** ^1^ School of Biotechnology Key Laboratory of Industrial Biotechnology (Ministry of Education) Jiangnan University Wuxi, Jiangsu 214122 China

## Abstract

Nattokinase (NK) is an important serine‐protease with direct fibrinolytic activity involving the prevention of cardiovascular disease as an antithrombotic agent. Dozens of studies have focused on the characterization of intrinsic novel promoters and signal peptides to the secretory production of recombinant proteins in *Bacillus subtilis*. However, intrinsic genetic elements have several drawbacks, which cannot mediate the production of NK to the desired level. In this study, the genetic elements, which were used to overproduce the recombinant secretory NK, were rationally modified in *B. subtilis* in a stepwise manner. The first step was to select a suitable signal peptide for the highly efficient secretion of NK. By comparison of the secretory levels mediated by two different signal peptides, which were encoded by the genes of a minor extracellular protease *epr* (SP_epr_) and cell‐wall associated protease *wapA* (SP_wapA_), respectively, SP_wapA_ was verified as the superior secretory element. Second, P04, which was a synthetic promoter screened from an array of mutants based on the promoter cloned from the operon of a quorum‐sensing associated gene *srfA* (P_srfA_), was paired to SP_wapA._ The secretory level of NK was obviously augmented by the combination of these two genetic elements. Third, the cis‐acting element CodY‐binding sequence positioned at the 5′UTR was deleted (yielding P08), and thus the secretory level was significantly elevated. The activity of NK, which was defined as fibrinolytic units (FU), reached to a level of 270 FU ml^−1^. Finally, the superior genetic element composed of P08 and SP_wapA_ was utilized to overproduce NK in the host *B. subtilis* WB800, which was able to produce the secretory NK at 292 FU ml^−1^. The strategy established in this study can not only be used to overproduce NK in *B. subtilis* but also might be a promising pipeline to modify the genetic element for the synthetic secretory system.

## Introduction

Nattokinase (EC 3.4.21.62, NK), a profibrinolytic serine protease originally extracted from a Japanese food called natto, is a member of the subtilisin family and possesses a strong activity of fibrin degradation. The enzyme is composed of 275 amino acids with a molecular weight of 27.7 KDa in its mature form and contains no disulfide bonds (Urano *et al*., 2001). NK is synthesized in a precursor form comprising a signal peptide and a pro‐peptide. These two parts are joined to the *N*‐terminus of the mature polypeptide (Nakamura *et al*., 1992). Compared to other fibrinolytic enzymes (urokinase, t‐PA and streprokinase), NK has the advantage of no side effects, low cost and long lifetime, has the potential to be used as a drug for treating cardiovascular disease and serves as a functional food additive (Cai *et al*., 2017a,b; Weng *et al*., 2017).

An array of NK‐producing *Bacillus* strains was originally screened for producing NK under naturally fermented conditions (Inatsu *et al*., 2006; Wang *et al*., 2006; Wei *et al*., 2011; Kumar *et al*., 2014). In recent years, along with the cloning of the *aprN* gene from *Bacillus* natto, NK has been overexpressed in a variety of hosts, including *Bacillus subtilis*,* Escherichia coli* and *Lactococcus lactis* (Liang *et al*., 2007a,b; Chen *et al*., 2010; Degering *et al*., 2010; Nguyen *et al*., 2013; Guan *et al*., 2016a,b). Although NK from a Douchi‐isolating *B. subtilis* YF38 has been successfully overexpressed in *E. coli* and transported to the periplasmic space, the yield of total enzyme was only 49 mg per litre of culture. The induced gene expression in *E. coli* requires isopropyl‐β‐D‐thiogalactopyranoside (IPTG) to activate the production of NK, which is potentially hazardous to food safety. *B. subtilis* is a soil‐derived bacterium and is generally regarded as safe. *B. subtilis* can naturally secrete heterologous proteins into the extracellular compartment, and thus this host has been broadly explored for the overproduction of numerous industrial and pharmaceutical proteins (Liu *et al*., 2013a,b). *Bacillus*, as well as *Lactococcus*, has been explored to overproduce recombinant NK as a workhorse (Liang *et al*., 2007a,b; Wu *et al*., 2011; Wei *et al*., 2015; Guan *et al*., 2016a,b).

To enhance the secretory capability and final yield of recombinant NK in *B. subtilis*, several strategies employing multiple biological and process engineering methods have been developed, including promoter engineering, signal peptide screening and optimization of fermentation process (Chen *et al*., 2010; Cho *et al*., 2010; Degering *et al*., 2010; Kwon *et al*., 2011; Wu *et al*., 2011; Guan *et al*., 2016a,b). Genetic engineering of the promoter has great potential for the augmentation of NK expression. By introducing the entire T7 system commonly used in the recombinant *E. coli* to the genome of *B. subtilis*, an IPTG‐induced protein expression system has been fabricated and applied to the overproduction of NK (Chen *et al*., 2010). Although this novel IPTG‐driven gene expression system successfully produced heterologous proteins, including NK, it is likely that this system can only be utilized at a rather small scale, as the inducer is too expensive to be used on a larger scale. Therefore, engineering a stronger constitutive promoter and chemical inducer‐free promoter are two fundamental promising methods in *B. subtilis* for the large‐scale fermentation of recombinant proteins. Moreover, the signal peptides directing the overexpressed proteins to the extracellular space were also largely screened, and diverse Sec‐dependent signal peptides were well equipped to suitable promoters. These combined genetic elements have produced higher levels of NK (Liang *et al*., 2007a,b; Wu *et al*., 2011; Wei *et al*., 2015).

The directed evolution of bacterial promoters and Sec‐dependent signal peptides is a practical method to augment the yields of heterologous proteins (Leavitt and Alper, 2015). Two areas, the −10 box and −35 box, located at the bacterial promoter, are critical to the level of gene transcription (Lee *et al*., 2012; Blazeck and Alper, 2013). According to some reports, modification of these two elements may be a feasible strategy to augment the transcription levels of downstream heterologous genes (Phan *et al*., 2012; Cheng *et al*., 2016). However, it is difficult to equip a universal signal peptide with a specific promoter, by which the recombinant system harbouring the two combined genetic elements could express and secrete the heterologous element at the desired level. To overcome the problem of efficient compatibility between these two genetic elements for the secretion of heterologous proteins in *Bacillus*, some studies have been selected and screened for diverse signal peptides from *B. subtilis* and *B. licheniformi* using a protease from a different strain from the two hosts (Degering *et al*., 2010). Importantly, the effects of several Sec‐dependent signal peptides on the production of NK in recombinant *B. licheniformis* were systematically compared and revealed that the expression and secretion of NK varied according to the utilization of various signal peptides (Wei *et al*., 2015). There are other examples showing the similar principle in different model proteins, such as aminopeptidase, which were extracellularly produced in *B. subtilis* (Guan *et al*., 2016a,b). According to these experimental facts, it is more reasonable to infer that different proteins must be equipped with an optimized combination of promoter and signal peptide to achieve the desired secretory level.

Therefore, in this study, two Sec‐dependent signal peptides were selected, and the secretion levels of NK were compared in *B. subtilis*. The more efficient signal peptide was combined with an array of engineered promoters derived from the auto‐inducible promoter P_srfA_. The superior promoter‐signal‐combinatorial genetic element yielded a higher expression level and stronger secretory capability. These results provide a secretory NK system with enhanced functionality and show that the secretion of heterologous proteins in *B. subtilis* requires comprehensively combined genetic elements.

## Results and discussion

### Selection of a suitable signal peptide for the secretion of recombinant nattokinase

**Figure 1:**
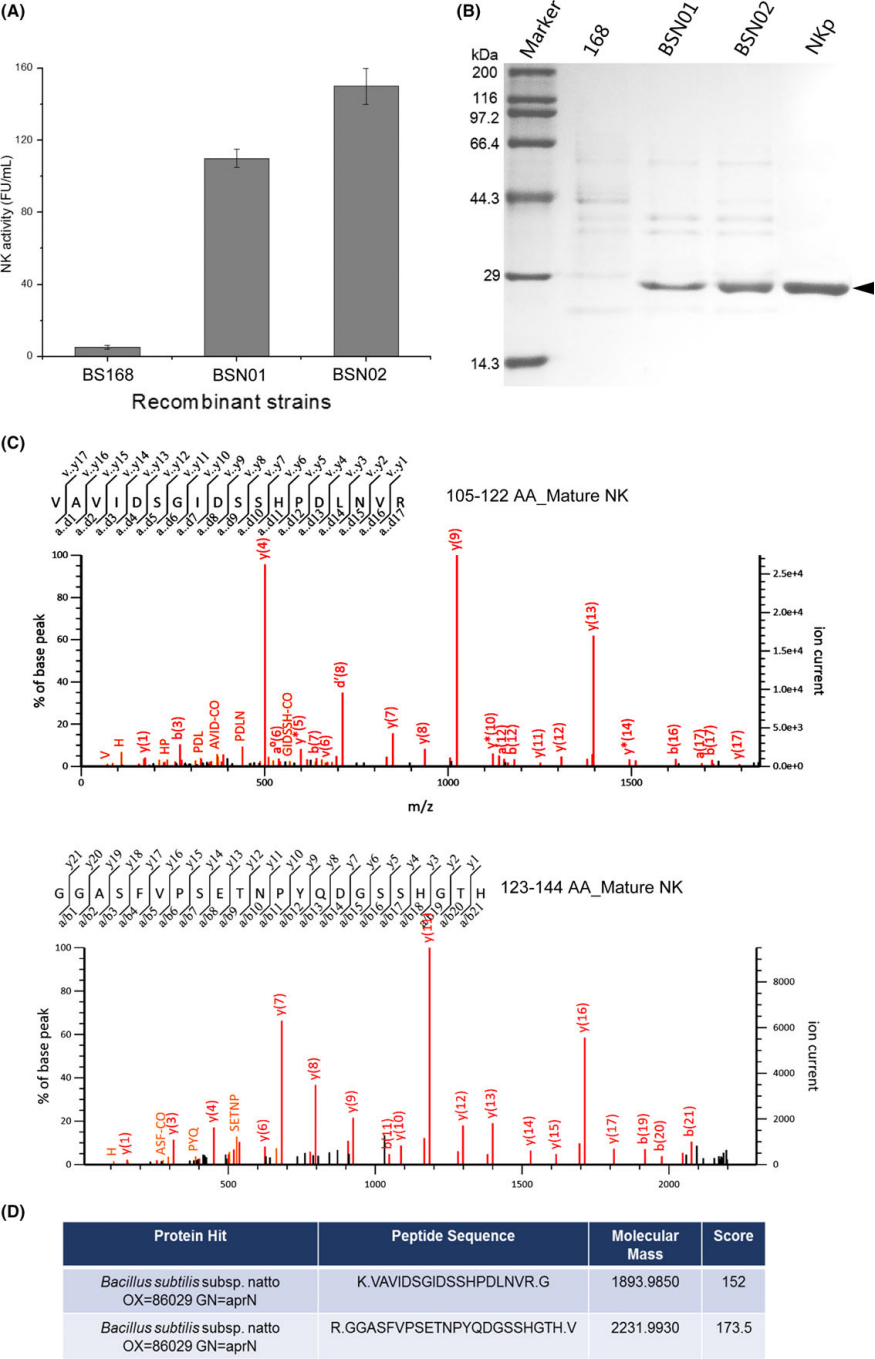
Selection of prior signal peptide for overproduction of secreted nattokinase in *Bacillus subtilis*. A. Enzymatic production of secretory nattokinase in recombinant *B subtilis* BSN01, BSN02 after 36 h of culture, directed by SP_epr_ and SP_wapA_, respectively. Background production of NK was determined by enzymatic activity in wild‐type *B. subtilis* 168. All experiments were independently performed in triplicate, and the values are shown as the mean ± SD. B. SDS‐PAGE analysis was used to detect secretory production levels of nattokinase in culture supernatant from BSN01 and BSN02 after 36 h of culture. Lane M denotes protein molecular broad marker. Lane NKp on the far‐right lane denotes purified NK, which verifies position of NK band on SDS‐PAGE. Arrow indicates NK protein band. C. The two sets of profiles of peak intensity of NK analysed by MALDI‐TOF/TOF MS. The two sequences of the peptide segment of NK are shown on the top of the panels and the corresponding position to the mature NK is also shown aside. D. The information of the sequence alignment by the Mascot. The names of the protein hits along with the peptide sequences, the corresponding molecular mass and the ion scores are shown, which fully authenticate that the band indicated on the SDS‐PAGE is NK.

The recombinant nattokinase can be transported across the extracellular membrane, mediated by either its native or heterologous Sec‐dependent signal peptides in recombinant *B. subtilis*,* L. lactis* and *E. coli* (Liang *et al*., 2007a,b; Wu *et al*., 2011), suggesting that recombinant NK has broad compatibility with diverse signal peptides for transportation and secretion in different hosts. Therefore, it is essential to explore various signal peptides to efficiently secrete recombinant NK in suitable hosts. In our previous study, an array of Sec‐dependent native signal peptides from *B. subtils* was utilized to overproduce aminopeptidase in recombinant *B. subtilis* 168. Based on the previously reported data of the secretome of *B. subtilis*, the signal peptides epr (SP_epr_) and wapA (SP_wapA_) displayed relatively high abundance at 0.883 and 4.45, respectively, while most of the approximately 80 characterized signal peptides showed abundance below 0.5 (Antelmann *et al*., 2006). Thus, the two signal peptides were selected as two potential candidates to validate the secretory efficiency and compatibility of the transportation of recombinant NK in *B. subtilis*. The recombinant strains BSN01 and BSN02, harbouring the constructs SP_epr_ and SP_wapA_, respectively, were fused upstream of mature NK and compared to validate the secretory levels. The produced enzymatic activities of the two strains were compared to determine which fusion produced a higher yield of secretory NK in the recombinant system (Fig. 1A). The enzymatic activity produced by BSN01 and BSN02 after 36‐h culture reached 110 ± 5 and 150 ± 12 FU ml^−1^, respectively. The background secretory level of NK in blank *B. subtilis* 168 was relatively low, but secretory NK with BSN02 was approximately 1.4‐fold higher than with BSN01, indicating that the secretion of NK mediated by SP_wapA_ has higher efficiency than with SP_epr_. These results were further verified by SDS‐PAGE analysis (Fig. 1B, left panel). The purified NK served as the reference to show the exact position of NK via SDS‐PAGE (Fig. 1B, right panel).

Furthermore, to fully authenticate the band of overproduced NK observed on SDS‐PAGE, the band of NK on SDS‐PAGE shown in Fig. 1B was cut off and subjected to MALDI‐TOF/TOF MS analysis. After alignment by Mascot, the positive hits that had the significance of *P *<* *0.05 were analyzed. The results were shown in Fig. 1C and D. The peak profiles of two segments of NK obtained from the MALDI‐TOF/TOF MS were shown in Fig. 1C. The sequences of the two segments best matched the segment of the mature NK at the position of 105–122 and 123–144. The sequence was shown to be the aprN (the NK protein) from *Bacillus subtilis* subsp. natto. These results fully supported the abovementioned conclusion. Interestingly, the secretory level of NK directed by SP_wapA_ in *B. subtilis* 168 was drastically higher than in recent reports, and NK was secreted by SP_epr_ in the recombinant *B. subtilis* WB600 and transported by the signal peptide S_sacC_ in recombinant *B. licheniformis* (Wei *et al*., 2015; Guan *et al*., 2016a,b). Therefore, these data show that the signal peptide SP_wapA_ has tremendous potential for highly transporting NK in *B. subtilis*. It is interesting to determine whether SP_wapA_ can continue directing the high secretion of more heterologous proteins.

### Determination and equipment of a stronger engineered promoter for SP_wapA_ to enhance expression of nattokinase

**Figure 2:**
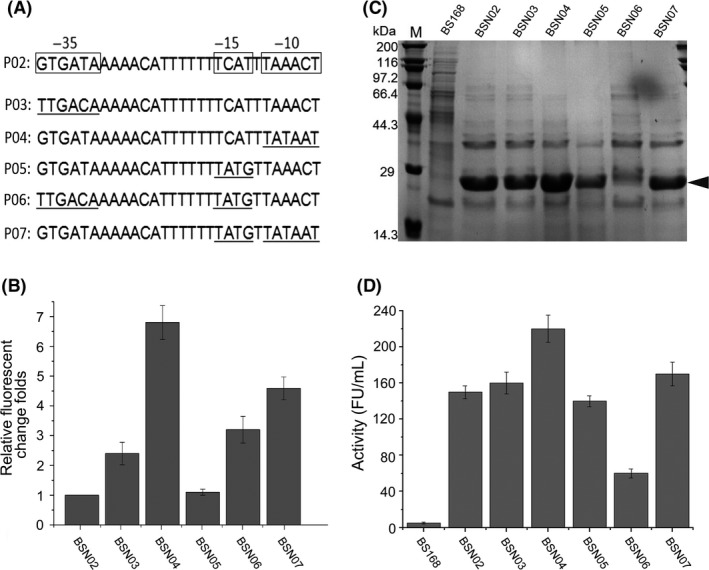
Selection and equipment of a prime engineered promoter for SP_wapA_ for highly secreting NK in *B. subtilis*. A. Schematic diagram exhibiting five mutants derived from native P_srfA_ P02. P03, P04, P05, P06 and P07 with mutated promoter, with different mutations at −10 box, −15 box and −35 box, which are underlined on the sequence. Rectangular box on P02 marked three boxes of core region on wild‐type P_srfA_. B. qRT‐PCR was performed to detect relative transcriptional levels of NK in BSN03, BSN04, BSN05, BSN06 and BSN07, triggered by P03, P04, P05, P06 and P07, respectively. Transcriptional level of BSN02 under control of native P02 was defined as control. All experiments were independently performed in triplicate. Data are shown as the mean ± SD. C. SDS‐PAGE analysis for detection of secretory production level of NK after 36 h of culture. M denotes the protein marker. Arrow indicates secreted NK. D. Measurement of enzymatic activities yielded in BSN03, BSN04, BSN05, BSN06 and BSN07, driven by P03, P04, P05, P06 and P07, respectively. Background level of secreted NK determined in BSN02. All experiments were independently performed in triplicate, and values are shown as the mean ± SD.

Transcription activity, translation initiation and secretory efficacy determine the ultimate productive levels of secretory recombinant proteins (van Dijl and Hecker, 2013; Liu *et al*., 2013a,b). To improve the expression levels of the recombinant proteins in bacterial hosts, promoter engineering is used to construct promoter variants to generate stronger transcription (Wu *et al*., 2011; Phan *et al*., 2012; Guan *et al*., 2016a,b). In our recent reports, a series of mutant promoters based on P_srfA_ was fabricated by semi‐rational design via combinatorial modification of the −10 box and the −35 box. The expression levels driven by these novel synthetic promoters were characterized by the reporter gene, and the compatibility to the target proteins was validated by testing the expression of amino peptidase (Cheng *et al*., 2016). However, it was still unclear whether higher expression levels of NK could be achieved using these promoter variants. To determine the superior synthetic promoter that was compatible to trigger SP_wapA_‐NK at high levels, five synthetic promoters, P03, P04, P05, P06 and P07 derived from native P_srfA_ (Fig. 2A), were fused upstream of the expression cassette SP_wapA_‐NK, yielding five recombinant strains: BSN03, BSN04, BSN05, BSN06 and BSN07. BSN02 harbouring the native P_srfA_ (P02) was set as the positive control. The transcriptional levels of NK in BSN03, BSN04, BSN05, BSN06 and BSN07, which were under the control of promoter mutants P03, P04, P05, P06 and P07, respectively, were measured and compared to BSN02. The data showed that the transcriptional level of NK in BSN04 was dramatically higher than the others, at 6.8‐fold higher than BSN02 (control), indicating that altering the −10 box of P_srfA_ to the consensus sequence can enhance the transcription level in *B. subtilis* (Fig. 2B). The expression levels of NK in these recombinant hosts were further verified at the translational level using SDS‐PAGE analysis (Fig. 2C). These results were consistent with those at the transcriptional level. To evaluate the quality of the secreted NK, the enzymatic activity produced by each of the six recombinant strains was also measured. The data showed that BSN04 produced the highest level of secretory NK after 36‐h culture, which reached to 220 ± 15 FU ml^−1^. Although the secretory production of NK in BSN03 and BSN07 was 160 ± 12 and 170 ± 14 FU ml^−1^, marginally higher than the positive control (BSN02), the produced levels of enzymatic activities driven both by P03 and P07 were obviously higher than for BSN02, which indicates that the modification of the −35 box alone and combinatorial modifications of the −10 box and the adjacent region −15 box positively regulated the strength of the P_srfA_, Moreover, the promoter strength of the variants P04 and P07 obviously showed that the −15 box has a substantial influence on the transcriptional strength of P_srfA_. It is important that P06, which was the combinatorial mutations at the −35 box and −15 box, was weaker than other synthetic promoters in this study (Fig. 2D). Notably, the mutant promoter mediating the highest level of overproduction of NK differs from that mediating the production of GFP compared to our previous reports (Cheng *et al*., 2016; Guan *et al*., 2016a,b). These data demonstrate that it is impractical to fabricate a universal promoter mediating equivalent expression levels for diverse target proteins since the expression of heterologous protein is determined by two genetic steps: transcription and translation. The promoter only controls the first step, which is highly associated with its strength (Schumann, 2007). Importantly, the translation step is regulated by the stability of mRNA, the RBS sequence and the secondary structure of the 5′‐UTR (Jurgen *et al*., 1998; Kozak, 1999; Osterman *et al*., 2013). Therefore, different heterologous proteins overexpressed in the recombinant *B. subtilis* should be equipped with differentially engineered promoters so that the expression level of the recombinant proteins can be maximized by the appropriate combinatorial promoter element and the target protein.

Elimination of negative regulation by CodY through deletion of the CodY‐binding site resulted in augmented NK production.

**Figure 3:**
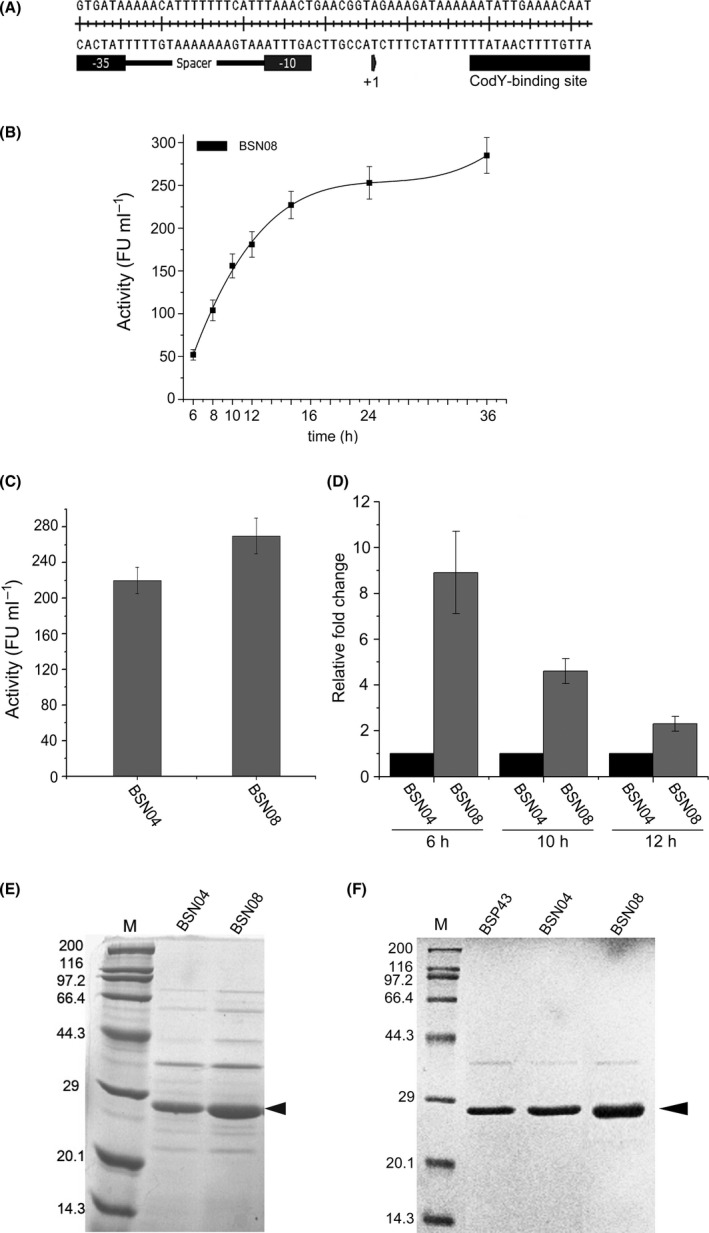
Elimination of negative transcription regulation by regulator CodY through deletion of cis‐acting element on P_srfA_ resulted in augmentation of secretory production of NK. A. Sketch of core region of P_srfA_ including CodY‐binding site. The +1 indicates transcription start site. The cis‐acting element CodY‐binding site on P_srfA_ is underlined. The −35 and −10 boxes are denoted by solid rectangular boxes. B. The time course‐dependent profile of secretory enzymatic activity of NK was determined in the BSN08 harbouring promoter with the CodY‐binding site deleted. All experiments were independently performed in triplicate, and the data are shown as the mean ± SD. C. Secretory enzymatic activities of NK produced by *B subtilis* BSN04, BSN08 after 36 h of culture. BSN08 harboured the mutant promoter P08 deficient in the CodY‐binding site. All experiments were performed in triplicate, and the values are shown as the mean ± SD. D. Differences in transcriptional level between P04 with CodY‐binding site and P08 without CodY‐binding site were determined by qRT‐PCR after culturing for 6, 10 and 12 h. All experiments were performed in triplicate, and the data are shown as the mean ± SD. E. SDS‐PAGE analysis for detection of secretory production level of nattokinase in BSN04 and BSN08 after 36 h of culture. Lane M is the protein molecular broad marker. Arrow indicates nattokinase. F. Secretory level of NK in BSP43, BSN04 and BSN08, driven by a commonly used strong promoter P43, P04 (with CodY‐binding site) and P08 (without CodY‐binding site), respectively, as detected by SDS‐PAGE analysis. SP_wapA_ was the secretory element in these two constructs. Samples were collected after 36‐h cultivation.

CodY is a global transcriptional regulator in *B. subtilis* that directly or indirectly controls dozens of genes. It is induced as bacterial cells make the transition from a rapid exponential phase to a stationary phase by sensing intracellular GTP levels (Ratnayake‐Lecamwasam *et al*., 2001; Molle *et al*., 2003). The parental promoter P_srfA_ used in this study, derived from the *srf* operon, is naturally regulated by CodY (Cosby *et al*., 1998; Bergara *et al*., 2003). The consensus CodY‐binding site has been identified in *B. subtilis*, which has a 15‐bp motif upstream of the CodY‐regulated promoters (Belitsky and Sonenshein, 2008). Since CodY acts predominantly as a repressor of transcription, it is reasonable to infer that the CodY‐binding site within the P_srfA_ tethers transcription in the recombinant *B. subtilis* system. It is critical to prevent CodY from tethering the transcription activation triggered by P_srfA_ and the corresponding mutants in the recombinant *B. subtilis* expression system to allow unrestrained transcription strength. To this end, the CodY‐binding site (AATATTGAAAACAAT) downstream of the transcription start site (+1) was deleted so that P_srfA_ did not suffer from tethering of CodY during the early exponential phase (Fig. 3A). The plasmid pBSN08 harbouring P08 mutant deficient in the CodY‐binding site was transformed in *B. subtilis* 168, yielding BSN08. The profile of the secretory activity of NK was measured at different culture times. The secreted NK depending on the time course prominently increased after 6‐h cultivation, and the rapid increase in secretory NK was continuous during the 16‐h cultivation (Fig. 3B). The secretory overproduction of NK mediated by P04 and P08 in BSN04 and BSN08, respectively, after 36‐h cultivation was measured to compare the influence of deficiency in CodY‐binding site on the overproduced level of NK. The NK produced in BSN04 and BSN08 was measured using the enzymatic activity assay and further verified by SDS‐PAGE analysis. The secretory yield of enzymatic activity produced by BNS08 reached 270 ± 17 FU ml^−1^ after 36‐h culture, which was 1.2‐fold higher than BNS04 (220 ± 15 FU ml^−1^) (Fig. 3C).

Furthermore, the transcriptional levels of NK in BSN04 and BSN08 after culture for 6, 10 and 12 h were measured by qRT‐PCR. The data showed that the transcriptional level of NK under the control of promoter without a CodY‐binding site displayed consistently higher expression than that with the cis‐activating factor, indicating that the CodY‐binding site within the promoter P_srfA_ regulates the expression of NK at the transcription level (Fig. 3D). The SDS‐PAGE analysis confirmed that the secretory amount of NK produced by BSN08 was much higher than in BSN04 (Fig. 3E).

To exhibit the superiority of the fabricated P08, a strong promoter P43 that has been commonly used in *B. subtilis* (Ying *et al*., 2012; Zhang *et al*., 2013), was employed to overproduce NK. SDS‐PAGE analysis of the secretion of NK mediated by SP_wapA_ showed that BSN08 harbouring P08 (with deleted CodY‐binding site) produced relatively higher secreted NK than P43 and P04 (Fig. 3F). These results demonstrate that P04 without tethering of negative regulation by CodY regulator may strengthen the transcription level. Although P_srfA_ has an atypical CodY‐binding site, without the 15‐bp consensus sequence (Belitsky and Sonenshein, 2008), the transcription of genes driven by P_srfA_ is still subject to the regulation of the CodY regulator. Recently, a study revealed that there are multiple copies of the CodY‐binding site, with a 6‐bp overlay positioned at some promoters in *B. subtilis* (Wray and Fisher, 2011). Thus, it is reasonable to infer that there might be other unknown or unidentified CodY‐binding sites at P_srfA_ that still function when P08 is working. Investigating how to thoroughly clean up restriction of transcription by the CodY regulator via promoter engineering should be a promising perspective for the further enhancement of the secretory production of NK in *B. subtilis*.

### The compatibility of diverse hosts to overproduction of nattokinase

**Figure 4:**
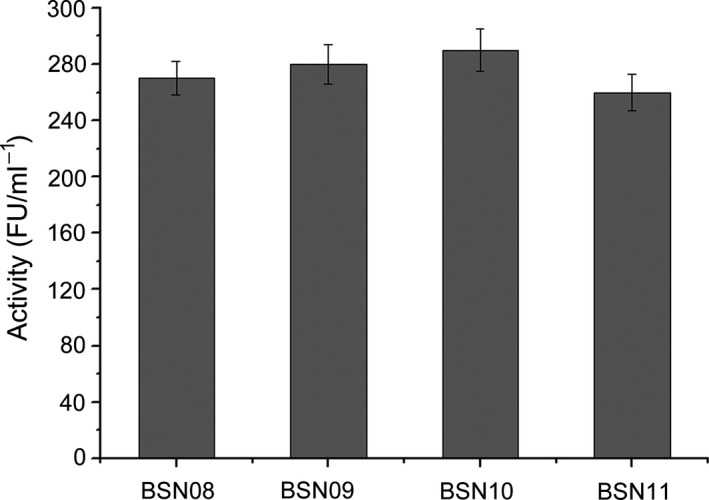
Comparison of secretory overproduction of NK in diverse *B. subtilis* strains. Secretory enzymatic activities of nattokinase produced by BSN08, BSN09, BSN10 and BSN11 after 36 h of culture compared to determine the superior host to be suitably combined with pBSN08. All experiments were performed in triplicate, and the values are shown as the mean ± SD.

To select the most suitable host to the secretory system, the production levels of secretory NK in three other *B. subtilis* 168‐derived hosts, WB600, WB800 and sigF (Guan *et al*., 2016a,b), were measured and compared according to enzymatic activity. The pBSN08 was transformed into WB600, WB800 and sigF, producing BSN09, BNS10 and BSN11, respectively. BSN08 served as the control. The data in the enzymatic assay exhibited the secretory production of NK in BSN08, BSN09, BSN10 and BSN11 at 272 ± 12, 281 ± 14, 292 ± 17 and 260 ± 14 FU ml^−1^, respectively (Fig. 4), indicating that WB800, which is deficient in the eight extracellular proteases, produces more secreted NK. This likely result from less protease degradation of NK by the extracellular components produced by this strain.

**Figure 5:**
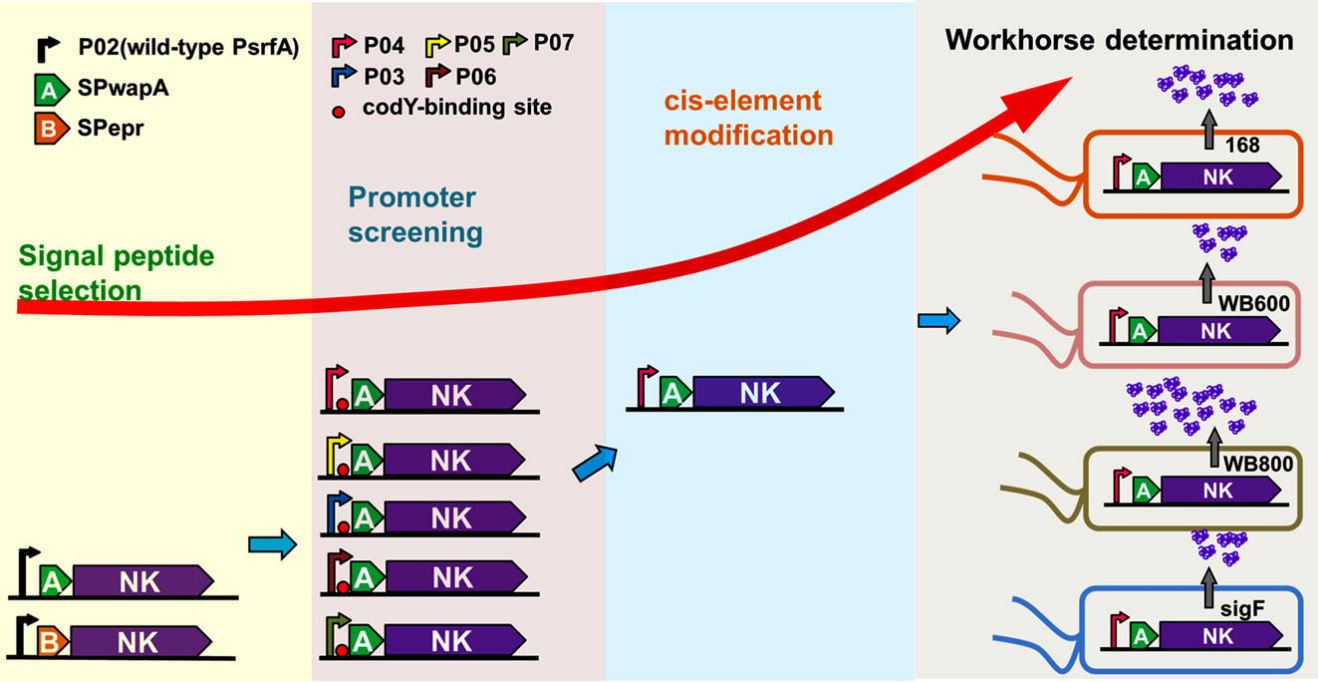
Flow chart recapitulates pipeline concept for combinatorial genetic modification. Four steps from the selection of signal peptide to the determination of the superior host were performed to modify the expression and secretion of recombinant NK in *Bacillus subtilis*. The superior signal peptide SP_wapA_ was first selected by comparison to SP_epr_, a highly efficient signal peptide. Then, an array of synthetic promoters derived from P_srfA_ was tested with SP_wapA_. P04 was verified as the best element combined with SP_wapA_. The combination of two genetic elements produced an elementary portion for the secretion of NK in *B. subtilis*. Accordingly, P04 was further engineered by deletion of the CodY‐binding site upstream of the translation start site. Finally, the most suitable host workhorse for high secretion of NK was determined. The legend denoting each part of the flow chart is shown in the diagram.

In summary, recombinant NK was overproduced and secreted via combinatorial modification of genetic elements in a stepwise manner in this study. The conception of this strategy was recapitulated and depicted in Fig. 5. Although some reports have focused on engineering genetic elements to enhance the secretory level of heterologous proteins, our study provides a promising pipeline for efficiently engineering a complex expression system composed of several biological parts, which must be synchronized for better performance.

## Experimental procedures

### Bacteria and cultural conditions

The bacteria used in this study are listed in Table 1. *Escherichia coli* JM109 was used as the host to clone the genes and propagate the recombinant plasmids. *Bacillus subtilis* 168, *B. subtilis* WB600 (deficient in six extracellular proteases) and *B. subtilis* WB800 (deficient in eight extracellular proteases) were used for the secretory expression of Nattokinase. Recombinant *E. coli* JM109 was cultured in Luria‐Bertani (LB) medium (Tryptone 10 g l^−1^, NaCl 10 g l^−1^, Yeast extract 5 g l^−1^, pH 7.0. Agar powder (20 g l^−1^). Wild‐type *B. subtilis* 168 *B. subtilis* WB600 and *B. subtilis* WB800 and the corresponding recombinant strains were cultured in Terrific Broth (TB) medium (yeast extract 24 g l^−1^, tryptone 20 g l^−1^, glycerol 4 ml l^−1^, KH_2_PO_4_, 0.17 M, K_2_HPO_4_, 0.72 M). The agar (2%, w/v) was added when the solid media were prepared. The media used for growing *B. subtilis* and *E. coli* harbouring the shuttle plasmids was supplemented with Kanamycin (5 μg ml^−1^) and Ampicillin (50 μg ml^−1^), respectively. All strains were cultured at 37°C and the cell density was determined by measuring the OD_600_ with a UV‐1800/PC spectrophotometer (MAPADA Instrument CO., Ltd., Shanghai, China).

**Table 1:** Strains and plasmids used in this study

Strains and plasmids	Relevant properties	References
Strains		
*Escherichia coli* JM109	*recA*1, *endA*1, *gyrA*96, *thi*‐1, *hsdR*17(r_k_^−^m_k_^−^), *e*14^−^(*mcrA* ^−^), *supE*44, *relA*1, Δ(*lac*‐*proAB*)/F′ [*traD*36, *proAB* ^+^, *lacI* ^q^, lacZΔM15]	Lab stock
*Bacillus subtilis* 168	*trpC2*	(Zeigler *et al*., 2008)
*B. subtilis* WB600	168, ΔnprE, ΔaprA, Δepr, Δbpr, Δmpr, ΔnprB	(Wu *et al*., 1991)
*B. subtilis* WB800	168, ΔnprE, ΔaprA, Δepr, Δbpr, Δmpr, ΔnprB, Δvpr, ΔwprA	(Wu *et al*., 2002)
*B. subtilis* sigF	168, ΔsigF	(Guan *et al*., 2016a,b)
BSN01	168, pBSN01(P_srfA‐_SP_epr_‐NK)	This study
BSN02	168, pBSN02(P_srfA‐_SP_wapA_‐NK)	This study
BSN03	168, pBSN03	This study
BSN04	168, pBSN04	This study
BSN05	168, pBSN05	This study
BSN06	168, pBSN06	This study
BSN07	168, pBSN07	This study
BSN08	168, pBSN08	This study
BSP43	168, pBSP43	This study
BSN09	WB600, pBSN08	This study
BSN10	WB800, pBSN08	This study
BSN11	ΔsigF, pBSN08	This study
Plasmids		
pMA09	*E. coli*‐*B. subtilis shuttle vector, Ap* ^r^ *Kan* ^r^ P_HpaII_	Lab stock
pMA0911	*E. coli*‐*B. subtilis shuttle vector, Ap* ^r^ *Kan* ^r^ P_HpaII_‐*NK*	Lab stock
pBSG03	P_srfA_‐*gfp*	(Guan *et al*., 2015)
pBSN01	pBSG03 with GFP replaced by SP_epr_‐NK	This study
pBSN02	pBSG03 with GFP replaced by SP_wapA_‐NK	This study
pBSN03	pBSN02 with P03	This study
pBSN04	pBSN02 with P04	This study
pBSN05	pBSN02 with P05	This study
pBSN06	pBSN02 with P06	This study
pBSN07	pBSN02 with P07	This study
pBSN08	pBSN04 with P08, deficient in CodY‐binding site	This study
pBSP43	pBSN02 derivative, P43 substitutes for P_srfA_	This study

### Genetic manipulation

*Escherichia coli* JM109 was used to propagate all recombinant plasmids. *Dpn*I (TaKaRa Bio Company, Dalian, China) was used to digest the templates after inverse PCR. PrimeSTAR^®^ DNA polymerase (TaKaRa Bio Company) was used for the PCR reaction. The DNA extraction kit was purchased from the TIANGEN Biotech (Beijing) Co., Ltd. (Beijing, China) and used for plasmid DNA isolation.

### Plasmid construction

The fragments of signal peptide *epr* and *wapA* were genetically fused upstream of the gene of mature NK and amplified using pMA0911 as a template with the primers P01‐up/P01‐down and P02‐up/P02‐down. After purification of the PCR products, each of the two fragments was used as megaprimers to substitute the gene of *gfp* on the plasmid pBSG03 by inverse PCR, yielding the recombinant plasmids pBSN01 and pBSN02 and harbouring the expression cassettes of SP_epr_‐NK and SP_wapA_‐NK, respectively. The two plasmids were employed to compare the secretory efficiency of NK directed by SP_epr_ and SP_wapA_.

To equip the suitable promoter for the highly secretory signal peptide for the overproduction of NK, a series of constructs harbouring the engineered P_srfA_‐derived mutants P03, P04, P05, P06, P07 and P08, which were previously been fabricated and characterized, were constructed by substitution of the wild‐type P_srfA_ using inverse PCR. The pBSN02 was used as the template, and the primer pairs P03‐up/P03‐down, P04‐up/P04‐down, P05‐up/P05‐down, P06‐up/P06‐down, P07‐up/P07‐down, P08‐up/P08‐down and Pp43‐up/Pp43‐down were utilized to construct pBSN03, pBSN04, pBSN05, pBSN06, pBSN07, pBSN08 and pBSP43 by inverse PCR, respectively (Tables 1 and 2). The recombinant plasmids were transformed by electroporation following a previously reported protocol (Meddeb‐Mouelhi *et al*., 2012), yielding the recombinant strains BSN03, BSN04, BSN05, BSN06, BSN07, BSN08 and BSP43.

**Table 2:** Oligonucleotides used in this study

Primers	Sequences
P01‐up	ATGACAATGATGAAAAACATGTCTTGCAAACTTGTTGTATCAGTCACTCTGTTTTTCAGTTTTCTCACCATAGGCCCTCTCGCTCATGCGGCCGGAAAAAGCAGTACAGAAAAGAAATACATTGTCGG
P01‐down	GCTTTTTCCGGCCGCATGAGCGAGAGGGCCTATGGTGAGAAAACTGAAAAACAGAGTGACTGATACAACAAGTTTGCAAGACATGTTTTTCATCATTGTCATACCTCCCCTAATCTTTATAAGCAGTG
P02‐up	TATGACAATGATGAAAAAAAGAAAGAGGCGAAACTTTAAAAGGTTCATTGCAGCATTTTTAGTGTTGGCTTTAATGATTTCATTAGTGCCAGCCGATGTACTAGCAGCCGGAAAAAGCAGTACAGA
P02‐down	CTTTTTCCGGCTGCTAGTACATCGGCTGGCACTAATGAAATCATTAAAGCCAACACTAAAAATGCTGCAATGAACCTTTTAAAGTTTCGCCTCTTTCTTTTTTTCATCATTGTCATACCTCCCCT
P03‐up	ACTTTTCACCCATTTTTCGGTTGACAAAAACATTTTTTTCATTTAAACTGAACGGTA
P03‐down	TTTAAATGAAAAAAATGTTTTTGTCAACCGAAAAATGGGTGAAAAGTTTCATGCGGG
P04‐up	ATAAAAACATTTTTTTCATTTATAATGAACGGTAGAAAGATAAAAAATATTGAAA
P04‐down	TTTTATCTTTCTACCGTTCATTATAAATGAAAAAAATGTTTTTATCACCGAAAAA
P05‐up	CGGTGATAAAAACATTTTTTTATGTTAAACTGAACGGTAGAAAGATAAAAAAT
P05‐down	CTTTCTACCGTTCAGTTTAACATAAAAAAATGTTTTTATCACCGAAAAATG
P06‐up	CATGAAACTTTTCACCCATTTTTCGTTGACAAAAACATTTTTTTATGTTAAACTGAACGGTAGAAAGATAAAAAATATTG
P06‐down	CTTTCTACCGTTCAGTTTAACATAAAAAAATGTTTTTGTCAACGAAAAATGGGTGAAAAGTTTCATGCGGG
P07‐up	CCCATTTTTCGGTGATAAAAACATTTTTTTATGTTATAATGAACGGTAGAAAGATAAAAAAT
P07‐down	CTTTCTACCGTTCATTATAACATAAAAAAATGTTTTTATCACCGAAAAATGGGTG
P08‐up	GAACGGTAGAAAGATAAAAGAATAAATAGCCAAAATTGGTTTCTTATTAG
P08‐down	CTAATAAGAAACCAATTTTGGCTATTTATTCTTTTATCTTTCTACCGTTC
PqNK‐up	GCATTTTTAGTGTTGGCT
PqNK‐down	TGGTATGGGTTTGTTTCA
Pq16s‐up	AAGTCCCGCAACGAGCGCAA
Pq16s‐down	TCGCGGTTTCGCTGCCCTTT
Pp43‐up	GTTTCGCCTCTTTCTTTTTTTCATGTGTACATTCCTCTCTTACC
Pp43‐down	AGGTGGCAGAGGGCAGGTTGATAGGTGGTATGTT

### Overproduction of nattokinase in recombinant strains

The single colonies of recombinant *B. subtilis* 168 and *B. subtilis* WB600 were picked and inoculated into test tubes with a working volume of 10‐ml TB and cultured at 37°C overnight with rigorous shaking. The overnight cultures were inoculated into 250‐ml conical flasks with a working volume of 30‐ml TB and the starting OD_600_ was modified to 0.05. The recombinant strains were cultured at 37°C with vigorous shaking for the appropriate times. The cultures were periodically sampled, and the produced NK was determined by SDS‐PAGE and enzymatic activity.

### qRT‐PCR analysis of transcriptional level of NK

qRT‐PCR was performed to determine the relative transcriptional levels of NK driven by the promoters with and without CodY‐binding site. The 16SrDNA gene was chosen as the internal reference. The recombinant strains BSN04 and BSN08 harbouring the promoters P04 (with CodY‐binding site) and P08 (without CodY‐binding site), respectively, were cultured in a test tube overnight at 37°C. Each culture was transferred to a new flask with 20‐ml LB medium, and the starting OD_600_ was adjusted to 0.05. The cultures were sampled after culturing for 6, 10 and 12 h prior to extracting the total RNA. Total RNA was extracted from cells using the RNAprep Pure Cell/Bacteria Kit (TIANGEN Biotech). Reverse transcription was performed with the PrimeScript TM RT reagent Kit with gDNA Eraser (Perfect Real Time) (Takara). Then, real‐time PCR was performed with SYBR^®^Premix Ex Taq TM II (Tli RNaseH Plus) (Takara) and a CFX96 Touch™ Real‐Time PCR Detection System (Bio‐Rad Laboratories, Inc., Hercules, CA, USA). The real‐time PCR programme was as follows: 95°C for 30 s, 40 cycles of 95°C for 5 s and 60°C for 30 s, 95°C for 10 s, 60°C for 30 s and increasing to 95°C to test the melt curve. The primer pairs PqNK‐up/PqNK‐down and Pq16s‐up/Pq16s‐down were used to detect the transcriptional level of NK and 16SrDNA, respectively (Table 2). Finally, the data were represented by the $2^{-\Delta \Delta C_{t}}$ method. All experiments were independently performed in triplicate, and the data were shown as the mean ± SD.

### Purification of nattokinase as reference marker

The single clone of recombinant *B. subtilis* 168 harbouring pBSG02 (BSN02) was inoculated into the test tube with 5‐ml TB medium. Afterwards, the recombinant strain was cultured for 12 h in the shaker at 37°C with rigorous shaking. Then, the appropriate volume of culture was transferred into a 500‐ml conical flask with TB medium of 60 ml. After 36‐h culturing at 37°C with rigorous shaking, the fermentation supernatant was separated from cells by centrifugation for 10 min at 8000 *g*. The collected supernatant was then subjected to the ammonium precipitation. The precipitant at gradient between 40% and 60% was collected prior to dialysis in Buffer A (20 mM phosphate‐buffered saline, pH 6.0) overnight. Afterward, the component was loaded onto the cation exchange column HiTrap SP HP (GE Healthcare Life Sciences, Beijing, China) and then purified using ÄKTA^TM^ pure protein purification system (GE Healthcare Life Sciences). A gradient elution method was performed using Buffer B (20 mM phosphate‐buffered saline, pH 6.0 with 1 M NaCl). The purity of the samples was detected by SDS‐PAGE. The sample with purity of 90% or higher was selected to serve as the reference marker.

### SDS‐PAGE analysis

SDS‐PAGE analysis was performed according to the previous report with minor modifications to accommodate the specific properties of NK (Guan *et al*., 2016a,b). The cultured recombinant *B. subtilis* strains were centrifuged at 4000 rpm for 15 min. Then, the supernatant of each strain was collected as the sample. Phenylmethanesulfonyl fluoride (final concentration was 1 mM) was used as a protease inhibitor preventing NK from degradation by serine‐proteases. The loading amounts of samples onto the gel were normalized by the OD_600_ of the bacterial cells.

### Matrix‐Assisted Laser‐desorption Ionization Time‐of‐Flight Mass Spectrometry (MALDI‐TOF MS) analysis for authentication of recombinant Nattokinase

The preparation of sample of recombinant NK for verification of the sequence was performed by MALDI‐TOF/TOF MS according to the previously reported experimental procedure (Savary and Vasu, 2012), which was somewhat modified depending on our requirement in this study. In brief, the fermented supernatant of BSG04 was firstly collected and run SDS‐PAGE. The target NK band on the SDS‐PAGE gel was then cut out prior to transferring to a 1.5‐ml centrifugation tube. Add 200 μl of 100 mM NH_4_HCO_3_ in 50% acetonitrile (ACN) to detain the sample piece followed by shake well at 37°C for at least 45 min (till the piece of gel was clear). Spin down briefly and discard the supernatant. Then, the gel was dehydrated by addition of 100 μl of anhydrous ACN at room temperature and incubating for 10 min so that the gel will shrink. Accordingly, briefly spin down and remove the solvent, then dry gel piece using the Digital Series SpeedVac™ Systems (ThermoFisher Scientific, Shanghai, China) for 10 to 15 min. For In‐gel digestion, 25 μl of trypsin (2.5–10 ng μl^−1^) was added to the sample and incubated at 4°C for 60 min. Then, 50 μl of 40 mM NH_4_HCO_3_ in 10% ACN was added into the reaction system, incubated overnight at 37°C. After digestion, 150 μl of ultrapure water was added to the gel piece and incubated for 15 min with frequent vortex. The supernatant containing the digested peptides was then transferred to a new centrifuge tube followed by addition of 50 μl of 5% trifluoracetic acid in 50% ACN and incubation at 37°C for 30 min. The supernatant was separated from the gel slice by centrifugation and then was dried using Digital Series SpeedVac™ Systems.

The appropriate amounts of the prepared peptides were mixed with 10 μl of freshly prepared α‐cyano‐4‐hydroxy‐cinnamic acid matrix (Sigma‐Aldrich, Shanghai, China). One microliter of target protein sample was added onto the well of a MALDI target plate prior to loading into the UltrafleXtreme™ MALDI‐TOF/TOF mass instrument (Bruker‐Daltonics, Billerica, MA, USA). Tryptic peptides were analysed in the positive ion reflector mode, and spectra were calibrated using Bruker peptide calibration standard II (Bruker‐Daltonics). For database alignment, an in‐house Mascot server (http://www.matrixscience.com) was used, and scores calculated by the Mowse scoring algorithm in Mascot (*P *<* *0.05) were considered as positive identifications.

### Determination of enzymatic activity of nattokinase

According to the Japan Natto Kinase Association (http://j-nattokinase.org/en/jnka_nattou_01.html), the activity of NK was defined as fibrinolytic units (FU) because this method has excellent quantitative performance using fibrin as the substrate. The activity of NK was measured and determined according to previously reported protocols (Guan *et al*., 2016a,b; Cai *et al*., 2017a,b). In brief, 0.7 ml of Tris–HCl (50 mM, pH 8.0) was first mixed with 0.2 ml of fibrinogen solution (0.72%, w/v). After incubation for 10 min at 37°C, 50 μl of thrombin (20 U l^−1^) was supplemented into the catalytic solution followed by thorough blending, after which the mixture was incubated for 10 min at 37°C. Finally, 100 μl of fermentation supernatant with appropriate dilution was added to trigger the catalysis. After 60 min of catalysis, 1 ml of trichloroacetic acid (TCA, 200 mM) was added to terminate the reaction. The catalytic solution was centrifuged at 15 000* g* for 15 min. The supernatant was collected to measure the absorbance at 275 nm (detecting the released tyrosine after hydrolysis) using a UV‐1800/PC spectrophotometer (MAPADA Instrument Co., Ltd.).

One unit (1 FU) is defined as the amount of enzyme that increases the absorbance of the filtrate at 275 nm by 0.01 per minute under the conditions specified in the procedure.

## Conflict of interest

None declared.
